# Translation, cross-cultural adaptation and validation of the Norwich Patellar Instability score for use in Brazilian Portuguese

**DOI:** 10.1590/1516-3180.2018.0393280119

**Published:** 2019-07-15

**Authors:** Lucas Simões Arrebola, Túlio Vinícius de Oliveira Campos, Toby Smith, André Lourenço Pereira, Carlos Eduardo Pinfildi

**Affiliations:** I PT, MSc. Doctoral Student, Department of Human Movement Sciences, Universidade Federal de São Paulo (UNIFESP), Baixada Santista Campus, Santos (SP), and Physical Therapist, Department of Physical Therapy, Instituto de Assistência Médica ao Servidor Público Estadual (IAMSPE), São Paulo (SP), Brazil.; II MD, PhD. Professor and Physician, Department of Orthopedics, Universidade Federal de Minas Gerais (UFMG), Belo Horizonte (MG), Brazil.; III PhD. Researcher, Nuffield Department of Orthopaedics, Rheumatology and Musculoskeletal Sciences, University of Oxford, Oxford, United Kingdom.; IV MD. Orthopedic Surgery Residency Student, Department of Orthopedics, Universidade Federal de Minas Gerais (UFMG), Belo Horizonte (MG), Brazil.; V PT, PhD. Professor, Department of Human Movement Sciences, Universidade Federal de São Paulo (UNIFESP), Baixada Santista Campus, Santos (SP), Brazil.

**Keywords:** Patellar dislocation, Knee, Surveys and questionnaires, Translations, Validation studies [publication type]

## Abstract

**BACKGROUND:**

The Norwich Patellar Instability (NPI) score is a tool for evaluating the impact of patellofemoral instability on joint function. It has not been translated or culturally adapted for the Brazilian population before.

**OBJECTIVE:**

This study had the aims of translating and culturally adapting the NPI score for use in Brazilian Portuguese and subsequently assessing its validity for this population.

**DESIGN AND SETTING:**

Translation, cross-cultural adaptation and validation study conducted at the State Public Servants’ Institute of São Paulo, Brazil.

**METHODS:**

Sixty patients of both sexes (aged 16-40 years) with diagnoses of patellar dislocation were recruited. The translation and cultural adaptation were undertaken through translation into Brazilian Portuguese and back-translation to English by an independent translator. Face validity was assessed by a committee of experts and by 20 patients. Concurrent validity was assessed through comparing the Brazilian Portuguese NPI score with the Brazilian Portuguese versions of the Lysholm knee score and the Kujala patellofemoral disorder score among the other 40 patients. Correlation analysis between the three scores was performed using Pearson correlation coefficients with significance levels of P < 0.05.

**RESULTS:**

The Brazilian Portuguese version of the NPI score showed moderate correlation with the Brazilian Portuguese versions of the Lysholm score (r = -0.56; 95% confidence interval, CI: -0.74 to -0.30; P < 0.01) and Kujala score (r = -0.57; 95% CI: -0.75 to -0.31; P < 0.01).

**CONCLUSION:**

The Brazilian Portuguese version of the NPI score is a validated tool for assessing patient-reported patellar instability for the Brazilian population.

## INTRODUCTION

Patellofemoral instability is characterized by episodes of subluxation and dislocation of the patellofemoral joint. It mainly affects young individuals of both sexes, with predominance in females. It accounts for approximately 3% of all injuries involving the knee joint.^1^ The risk factors that have been identified include: trochlear dysplasia, lateral patellar tilt > 20º, patellar height ratio > 1.2 according to the Caton-Deschamps index, tibial tuberosity to trochlear groove (TT-TG) distance > 16 mm, skeletal immaturity at the first episode of dislocation and history of contralateral patellar dislocation.^2^^,^^3^

Treatment for patellofemoral instability may be surgical or conservative, depending on the number of episodes of dislocation and anatomical risk factors. No consensus has been reached regarding which method is better, in terms of function, quality of life and number of recurrences.^4^^,^^5^^,^^6^

Outcome measurements can be used to determine functional performance and to aid in decision-making on treatment options. Currently, the outcome measurements that are used for assessing people with knee disorders include the Fulkerson patellofemoral score,^7^ the International Knee Documentation Committeeform,^8^^,^^9^ the Lysholm kneescore,^10^^,^^11^ the Kujala patellofemoral disorder score^12^^,^^13^ and the Norwich Patellar Instability (NPI)score.^14^ Of these, only the NPI score was designed specifically for people with patellofemoral instability. Nevertheless, all of these measurements *except* the NPI score have been translated and culturally adapted for the Brazilian population. The NPI score shows moderate inverse correlation with the Kujala patellofemoral disorder score and the Lysholm knee score (rho = -0.66 to -0.54; P < 0.05) and has high internal consistency (Cronbach’s alpha = 0.93).^14^

Since the NPI score has not been translated or culturally adapted for the Brazilian population, and since this is the only score specifically designed for individuals with patellofemoral instability, the aims of the present study were firstly to translate and culturally adapt the NPI score for use in Brazilian Portuguese and secondly to assess its validity for the Brazilian population.

## METHODS

### Ethical considerations

This study was approved by the research ethics committee of the State Public Servants’ Institute of São Paulo on August 16, 2018 (approval number: 2.825.402). All participants signed an informed consent form or an assent form, depending on their age.

### Procedures

#### Translation and cultural adaptation

The translation and cultural adaptation of the NPI score followed the procedure proposed by Price et al.^15^ The original English version of the NPI score was translated into Brazilian Portuguese by a bilingual expert certified translator who had no prior knowledge of the score. The Brazilian Portuguese version was then sent to another bilingual expert certified translator who independently back-translated the score into English without access to the original score. A multidisciplinary committee composed of two orthopedic knee surgeons and one physical therapist was responsible for comparing the Brazilian Portuguese translation of the original version with the back translation, to verify the semantics and idiomatic and cultural equivalence.

**Figure 1.**
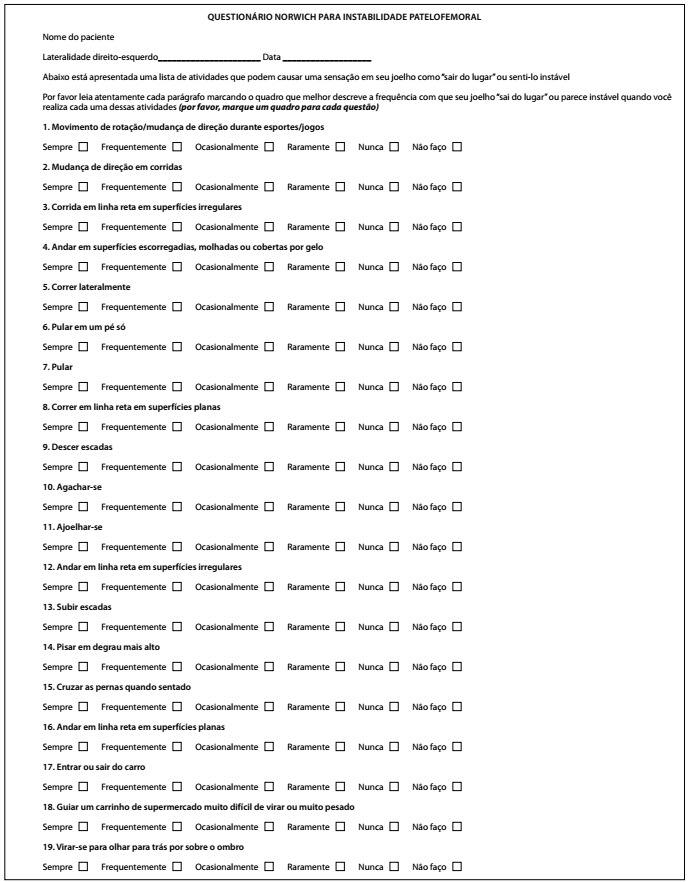
Translated and validated Brazilian Portuguese version of the Norwich Patellar Instability score.

**Figure 2.**
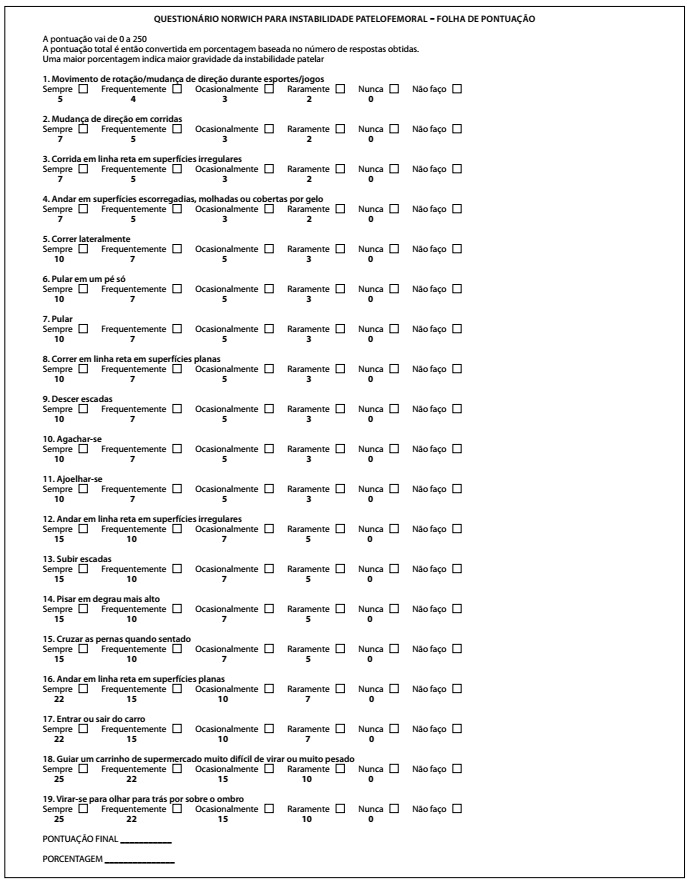
Score sheet of the Norwich Patellar Instability score translated into Brazilian Portuguese.

The NPI score consists of 19 questions relating to the perception of instability among subjects with histories of patellofemoral instability in sports and activities of daily life. It is scored from 0 (slightest sensation of instability) to 250 (greatest sensation of instability). The Brazilian Portuguese version consists of two parts: the first is the patient-completed questionnaire (Figure 1), and the second is a scoring sheet, which is used by a researcher to assign scores for each response, to determine the final score (Figure 2).

### Validity

#### Participants

Sixty participants (16 males; 44 females; mean age 20.85 years) were recruited from an orthopedic specialty outpatient clinic at the State Public Servants’ Institute of São Paulo. All consecutive patients admitted were invited until we had 60 participants, and they had the same cultural/social background. Eligible participants were required to have a documented episode of unilateral or bilateral patellar dislocation. All participants were required to present with two of the following clinical signs of patellofemoral instability: positive apprehension test, tenderness of the medial retinaculum on palpation or reported patellar instability on rotation or knee extension activities. Participants were excluded if they had previously experienced meniscal, cruciate or collateral ligament injury of the knee, history of hip, knee or ankle osteoarthritis, and if they reported a previous lower limb fracture or had undergone spinal or lower limb surgery irrespective of the surgical indication.

The pre-final version of the Brazilian Portuguese NPI score was piloted with 20 individuals of the 60 participants who had been diagnosed with patellar dislocation. This was used to evaluate their understanding of each item of the score. Once the Brazilian Portuguese NPI score version had been developed, the other 40participants with patellar dislocation were invited to the next phase of the study, to assess the concurrent validity of the score. Theparticipants filled out the questionnaire in person and without any assistance.

Concurrent validity was assessed by comparing the NPI score with the Brazilian Portuguese versions of the Lysholm knee score^11^ and the Kujala patellofemoral disorder score.^13^ The Lysholm knee score^10^ was created with the purpose of assessing symptoms of ligament injury and knee-related instability. It consists of eight closed questions with scores for each one. Its final score ranges from zero to 100, such that higher scores indicate that the patient is in better condition.^16^ The Kujala patellofemoral disorder score^12^ consists of 13 closed questions relating to the function of the knee joint, and it is directed towards patients with a history of patellofemoral joint involvement (pain and dysfunction). Its final score also ranges from zero to 100, such that higher scores indicate that the patient is in better function.

### Statistical analysis

The descriptive data were represented by the mean (with standard deviation). The assumption of normality was evaluated through visual inspection of the histogram and using the Shapiro-Wilk test. This showed that symmetrical distribution was present for all the data analyzed. The Pearson correlation coefficient was used to analyze the correlation between the NPI score, Lysholm knee score^11^ and Kujala patellofemoral disorder score,^13^ with an alpha error of P < 0.05. All data were presented with their 95% confidence intervals (CI). The statistical analysis was performed using the R software, version 3.4.4 for Windows (R Foundation, Vienna, Austria).

## RESULTS

The 40 participants with atraumatic patellar dislocation who participated in the validation process answered all the items of the questionnaires. Their demographic characteristics and score results are presented in Table 1.

**Table 1.** Characteristics of the participants

Variable	Mean (standard deviation)
Age (years)	20.22 (6.55)
Height (m)	1.67 (0.09)
Weight (kg)	64.72 (14.72)
Body mass index (kg/m²)	23.04 (4.39)
Number of episodes of dislocation	3.02 (2.27)
Age at the first episode of dislocation (years)	14.52 (4.41)
Lysholm knee score	59.65 (19.18)
Kujala patellofemoral disorder score	66.00 (14.83)
Norwich Patellar Instability score	96.02 (51.33)

m: meter; kg: kilograms; kg/m^2^: kilograms/square meter.

Table 2 shows the questions of the original NPI score and of the translation into Brazilian Portuguese. Figures 1 and 2showthe translated and validated Brazilian Portuguese version of the NPI score and the score sheet.

**Table 2.** Original and translated versions of the Norwich Patellar Instability score

Original version	Translated version
1. Twisting/changing direction during sports/games	1. Movimento de rotação /mudança de direção durante esportes / jogos
2. Changing direction when running	2. Mudança de direção em corridas
3. Running in a straight line on *uneven* surfaces	3. Corrida em linha reta em superfícies irregulares
4. Walking on slippery, wet, or icy surfaces	4. Andar em superfícies escorregadias, molhadas ou cobertas por gelo
5. Running sideways	5. Correr lateralmente
6. Hopping	6. Pular em um pé só
7. Jumping	7. Pular
8. Running in a straight line on *even* surfaces	8. Correr em linha reta em superfícies planas
9. Going downstairs	9. Descer escadas
10. Squatting	10. Agachar-se
11. Kneeling	11. Ajoelhar-se
12. Walking in a straight line on *uneven* surfaces	12. Andar em linha reta em superfícies irregulares
13. Climbing stairs	13. Subir escadas
14. Stepping onto or over a high step	14. Pisar em degrau mais alto
15. Crossing your legs when sitting	15. Cruzar as pernas quando sentado
16. Walking in a straight line on *even* surfaces	16. Andar em linha reta em superfícies planas
17. Getting into or out of a car	17. Entrar e sair do carro
18. Turning a heavy trolley round a supermarket aisle	18. Guiar um carrinho de supermercado muito difícil de virar ou muito pesado
19. Turning to look over your shoulder	19. Virar-se para olhar para trás por sobre o ombro
Always	Sempre
Often	Frequentemente
Sometimes	Ocasionalmente
Rarely	Raramente
Never	Nunca
Do not do	Não faço

The Brazilian version of the NPI score showed moderate correlation with the Brazilian Portuguese versions of the Lysholm knee score^11^ (r = -0.56; 95% CI: -0.74 to -0.30; P < 0.01) and the Kujala patellofemoral disorder score^13^ (r = -0.57; 95% CI: -0.75 to -0.31; P < 0.01). These results are summarized in Table 3.

**Table 3.** Correlation between the Norwich Patellar Instability score and alternative and similar instruments

Score	Pearson correlation coefficient	95% confidence interval	P-value
Lysholm knee score	-0.56	-0.74 to -0.30	< 0.01
Kujala patellofemoral disorder score	-0.57	-0.75 to -0.31	< 0.01

## DISCUSSION

This study demonstrated the translation, cultural adaptation and validation of the NPI score for use in the Brazilian population and its correlation with the Brazilian versions of the Lysholm knee score and the Kujala patellofemoral disorder score.

The translation and cultural adaptation of the NPI score followed the procedure proposed by Price et al.^15^ This procedure was adapted from Guillemin et al.,^18^ Bullinger et al.^19^ and Beatonetal.^20^ This procedure was used because: (1) patellofemoral instability comprises only 2%-3% of all knee injuries^1^ and, therefore, the affected individuals constituted a rare population; and (2) several authors have successfully used this procedure in other translation, validation and cultural adaptation processes.^21^^,^^22^^,^^23^

The Kujala patellofemoral disorder score^12^ and the Lysholm knee score^10^ are among the scores most used for evaluation of patellofemoral dysfunction in studies aiming to evaluate the efficacy of treatments for this condition.^24^^,^^25^^,^^26^ Both of these scores contain only a single item on knee instability, and only the first of them has an item on patellofemoral instability. However, this latter item only presents low correlation with the NPI score.^14^ This situation makes it difficult to accurately quantify the effect of these treatments on patients with patellofemoral instability and to adequately follow up the population.

Development of the NPI^14^ score has been found to be extremely important for adequate assessment of therapies for individuals with patellofemoral instability. It is currently the only tool available for this purpose. The NPI^14^ score consists of 19 questions that were based on a previous study that had aimed to assess which activities cause greater sensation of instability in these patients.^17^ Translation and validation of this score for Brazilian populations are important for development of studies in this country, including multicenter studies, and for extrapolation of the results thus obtained for use in clinical practice.

The results obtained from the present study regarding validation were similar to the findings previously reported^14^ from the development of the NPI score. That study also reported that there was a moderate correlation between the NPI score and the Lysholm knee score and the Kujala patellofemoral disorder score.^14^ As in the earlier study, the findings from the present study can be explained through the relationship between the NPI score and patellofemoral joint disorders and between this score and general knee instability. However, we hypothesize that a strong correlation between these instruments could not be observed in both studies because only the NPI score was developed specifically to assess cohorts with patellar instability.

Although the cohorts used in the two studies were different (such that in the earlier study, only individuals who were surgically managed were recruited), the results regarding validity were very similar. This suggests that the NPI score can be used for both conservatively and surgically managed patellar instability patients.

The most notable limitation of this study was that the responsiveness of the NPI score, i.e. the capability of the instrument to detect changes in the progression of a disease,^27^ was not assessed. Further studies are warranted, to assess the reliability, responsiveness and floor and ceiling effects of the Brazilian Portuguese version of the NPI score, and to establish its minimal clinically important difference (MCID). Establishment of the MCID would be particularly helpful for evaluating patient-reported outcomes, for guiding clinical practice and, ultimately, for enabling more optimally directed patient care.

Based on the findings from the present study, the Brazilian Portuguese version of the NPI score was satisfactorily translated. It proved to be a valid tool for use in research and clinical practice, in following up patients with patellofemoral instability.

## CONCLUSION

The NPI score has now been translated and culturally adapted and has been demonstrated to have validity for use in Brazilian Portuguese. Following this, the NPI score may now be considered for use within clinical and research practice, to aid in assessment and decision-making for individuals with patellofemoral instability.
